# Correlates of Intra-Household ITN Use in Liberia: A Multilevel Analysis of Household Survey Data

**DOI:** 10.1371/journal.pone.0158331

**Published:** 2016-07-12

**Authors:** Stella Babalola, Emily Ricotta, Grace Awantang, Nan Lewicky, Hannah Koenker, Michael Toso

**Affiliations:** Health Communication Capacity Collaborative, Johns Hopkins Center for Communication Programs, Johns Hopkins University, Baltimore, MD, United States of America; Centers for Disease Control and Prevention, UNITED STATES

## Abstract

Malaria is a major cause of morbidity and mortality in Liberia. At the same time, insecticide-treated net (ITN) ownership and use remain low. Access is a key determinant of ITN use but it is not the only one; prior studies have identified factors that affect the use of ITNs in households with at least one ITN. These factors operate at the individual, household, and community levels. However, studies have generally not assessed the psychosocial or ideational determinants of ITN use. Using 2014 household survey data, this manuscript examines the socio-demographic, ideational, household, and community factors associated with household member use of ITNs in Liberia. Multilevel modeling was used to assess fixed effects at the individual, household, and community levels, and random effects at the household and cluster levels. The data showed significant residual clustering at the household level, indicating that there were unmeasured factors operating at this level that are associated with ITN use. The association of age with ITN use was moderated by sex such that men, older children, and teenagers were less likely to sleep under an ITN compared to women and children under five years old. Female caregivers’ perceived severity of malaria, perceived self-efficacy to detect a complicated case of malaria, and exposure to the “Take Cover” communication campaign were positively associated with ITN use by members of her household. The association with household size was negative, while the relationship with the number of ITNs was positive. Programs should seek to achieve universal coverage (that is, one ITN for every two household members) and promote the notion that everyone needs to sleep under an ITN every night. Programs should also seek to strengthen perceived severity of malaria and educate intended audience groups on the signs of malaria complications. Given the significance of residual clustering at the household level, interventions that engage men as heads of household and key decision-makers are relevant.

## Introduction

### Background

Malaria is endemic in Liberia, representing a major cause of morbidity and mortality, and a leading cause of outpatient attendance and in-patient deaths in 2014 [1]. Children and pregnant women are the most affected by the disease. Rapid diagnostic testing conducted as part of the 2011 Malaria Indicator Survey revealed that 45% of children aged 6–59 months had malaria; microscopy revealed a lower prevalence (28%) [2]. The microscopy test results indicated that malaria prevalence increased monotonically with age from 9.6% among children aged 6–8 months to 35.4% among those aged 48–59 months. Other factors associated with variations in malaria prevalence among children included rural residence, county of residence, mother’s education, and household wealth quintile.

Insecticide-treated nets (ITNs) provide a physical barrier between humans and mosquitoes; the insecticide used on the nets also repels and kills mosquitoes. ITNs have been shown to lead to significant reductions in parasite rates in children under five years old and all-cause child mortality [3–5]. Additionally, there is evidence that ITN use by a majority of the community provides some level of protection even to those who are not using them, as it helps to reduce overall malaria transmission [6].

The government of Liberia is committed to reducing the burden of malaria in the country as evidenced in the 2010–2015 National Malaria Strategic Plan, which is the most recent national guidance available for Liberia [1]. The strategic plan builds on the two previous national malaria strategic plans and articulates strategies designed to reduce malaria morbidity and mortality, and reduce the incidence of malaria by 2015. The strategic plan has specific targets and strategies focused on each of the following four strategic areas: malaria case management, intermittent preventive treatment in pregnancy (IPTp), integrated vector control (including use of ITNs and indoor residual spraying (IRS)), and behavior change. Additionally, the plan recognizes the need to strengthen the capacity of the National Malaria Control Program in the development, management, and evaluation of malaria programs. The strategies articulated in the strategic plan are geared towards increasing prompt and effective treatment of malaria in children under five years old, increasing the uptake of IPTp for pregnant women, increasing access to IRS and ITNs in households, and increasing ITN use by children and pregnant women. Regarding ITN use, one of the key objectives of the National Malaria Strategic Plan seeks to increase the use of long-lasting insecticide-treated nets (LLINs) to 80% among children and pregnant women by 2010 and sustain this level of use through 2015.

Results of the 2013 Demographic and Health Survey showed that 55% of households in Liberia had at least one ITN while only 22% of households had universal coverage [7]. Only 38% of children under five and 37% of pregnant women slept under an ITN on the night preceding the survey. In households owning at least one ITN, 63% of both groups slept under an ITN the previous night.

In order to provide optimum protection, ITNs must be used regularly by all members of a population. Reasons for ITN use differ, but include the protection they offer from nuisance mosquitoes, perceived density of mosquitoes in one’s surroundings [8–11], and quality of the ITN [12]. The reasons often provided for non-use of ITNs include perceived heat, perceived discomfort of sleeping under an ITN, perceived low mosquito density, torn or worn ITNs, perception that the ITN was not needed, and unavailability of ITNs (lack of access) [10, 13–15].

Access is a key determinant of ITN use but not the only one. Studies that have focused on the factors associated with ITN use have highlighted the roles of age [16–21], sex [16, 19, 21–22], and pregnancy status [17]. Other factors found to be associated with variations in ITN use include household size [21, 23], and socio-economic status [18, 24]. Studies have also found a positive relationship between exposure to social and behavior change communication (SBCC) messages and ITN use in various settings [15, 23, 25–28]. In addition to these socio-demographic and program exposure variables, it is important to understand the cognitive, emotional, and social interaction factors that influence health behaviors. In the case of ITNs, these factors include knowledge, personal beliefs in one’s ability to obtain and use ITNs properly, perceived threat, perceived ITN efficacy, interpersonal communication, perceived social support, and social norms. For example, possessing accurate malaria knowledge has been found to be a strong predictor of ITN use in some settings [23, 29–32]. Similarly, beliefs that malaria is not a serious problem (low threat) and that the ITN is not effective in preventing malaria (low response efficacy) can contribute to low ITN use [10, 33–34].

The combination of these cognitive, emotional, and social interaction concepts is called “ideation.” Ideation is a concept frequently used in the family planning and HIV community as a way to understand people’s readiness to adopt health protective behaviors [35–37]. Ideation has only recently been explored in the malaria literature as a way to understand how mass media campaigns and community mobilizers influence the number of ITNs per person in a household [26].

### Objectives

Using household survey data collected between March and April 2014, this study evaluates the role of socio-demographic characteristics, caregiver ITN ideation, household characteristics, and community factors in ITN use among household members. The tested hypothesis is that the factors affecting ITN use operate at individual, household, and community levels. What differentiates this study from most previous studies is its focus on the role of ideational characteristics and unmeasured factors operating at the household and community levels in intra-household ITN allocation.

## Methods

### Study Setting

The study took place in four counties of Liberia in 2014, just prior to rainy season: Bong, Cape Mount, Grand Kru and Rivercess. Malaria prevalence among children under five years old (measured by rapid diagnostic test) was generally high in these counties and varied from 41.6% (95% CI: 33.9–48.7) in Bong to 69.5% (CI: 50.9–83.3) in Grand Kru (secondary analysis of MIS 2011 data performed by lead author). The four counties represented two endemicity strata in Liberia: Bong and Rivercess which have a malaria prevalence below the national average, and Grand Kru and Cape Mount which have a malaria prevalence above the national average.

### Study Design and Procedures

The data presented were derived from a cross-sectional household survey conducted in Liberia in 2014 by the Johns Hopkins Center for Communication Programs. A total of 1200 households were randomly selected from the four counties. The sample size was adequate to detect a 10-percentage point difference in ITN use between the two malaria endemicity strata. Households were selected through a multistage process that involved randomly selecting enumeration areas (clusters) with a probability proportional to size and then households from each study county. Only households with at least one child under the age of five years were eligible for participation in the survey. In selected households, a woman with a child aged less than five years old was randomly selected for interview. Through the household questionnaire, ITN use information was collected for 6,463 household members. The data analyzed in this manuscript combined ITN use information for individual household members derived from the household questionnaire with female caregivers’ socio-demographic and ideational characteristics derived from the individual questionnaire.

### Data Analysis

The dependent variable evaluated in this study is defined as sleeping under an ITN on the night before the survey. We limit the analysis to individuals from households with at least one ITN, representing only a little over one third of the households.

We assessed the predictive value of fifteen independent variables measured at the individual, household, and community levels and defined as follows:

1. Age: Household members were divided into three age groups: 0–4 years, 5–17 years, and adults aged 18 years and above;
2. Sex of household member: Male or female;
3. Education level of the female caregiver interviewed in the household, assessed as none, primary, or post-primary;
4. Female caregiver’s perception about ITN use being the norm in their community: An ideational variable assessed through a question that asks respondents in how many households in their community do people sleep under an ITN. The response options were: All households, most households, at least half of the households, fewer than half households, or hardly any households. We distinguished between the women that responded “all” or “most” households and those that gave other responses;
5. Female caregiver’s perceived susceptibility to malaria: Another ideational variable assessed through seven Likert-scale attitudinal statements related to the likelihood of getting malaria. For example, “During the rainy season, I worry almost every night that someone in my family will get malaria”; and “Nearly every year, someone in this community gets a serious case of malaria”. We scored each of the seven items between -2 and +2, computed a total score which we then split it at zero to denote low versus high perceived susceptibility;
6. Female caregiver’s perceived severity of malaria: Measured through five Likert-scale attitudinal items. For example: “When someone I know gets malaria, I usually expect them to completely recover in a few days” (reverse-coded); and “Every case of malaria can potentially lead to death”. We scored each of the five items between -2 and +2, computed a total score which we then split it at zero to denote low versus high perceived severity of malaria;
7. Female caregiver’s perceived self-efficacy to recognize a complicated case of malaria;
8. Female caregiver’s knowledge about malaria prevention: Measured as mentioning at least one correct malaria prevention method while not mentioning any incorrect prevention methods;
9. Female caregiver’s perceived self-efficacy to prevent malaria in self and children.
10. Female caregiver’s exposure to the “Take Cover” malaria communication campaign. The “Take Cover” campaign used radio spots, print materials and community mobilization to promote ITN use. It was first launched in 2009; the radio materials were periodically rebroadcast until the time of the survey in 2014. We defined high level of exposure to the campaign as having heard of the program from two or more media or community sources;
11. Number of ITNs in household: Classified as one, two or more than two;
12. Household size: The number of usual residents of the household;
13. Household socio-economic status: Derived from household assets through principal components analysis and divided into quintiles;
14. Number of under-five children within the household: We divided the number into one, two, or three or more; and,
15. County of residence: That is, Bong, Cape Mount, Grand Kru or Rivercess.

Multilevel modeling is the main analytic method used in this manuscript. The survey data analyzed in this manuscript reflect a hierarchical nature with individuals nested within their household, which are, in turn, nested within their clusters or neighborhood. In nested data, individuals within the same units tend to be more similar than those from other units. Ordinary regression methods do not account for this clustering and tend to underestimate the standard errors of regression coefficients [38]. When survey data are nested, multilevel modeling is the indicated analytic approach as it helps to adjust for clustering. To further justify the use of multilevel modeling, we computed the design effect. A design effect greater than 2.0 indicates violations of the independence assumption [39]. The design effect in our data was 11.4. In other words, clustering in the data results in over eleven-fold reduction in effective sample size compared to what would have occurred with simple random sampling.

We estimated three-level mixed effects models with fixed effects at the individual, household, and county levels, and random effects at the household and cluster levels. We estimated two models: an empty model without covariates to assess if there was significant group-level variation in ITN use, and the full model that included variables measured at the individual, household, and county levels. In multilevel models, a small number of groups and small group sizes have been documented to bias the efficiency of multilevel random parameter estimates downwards [39–41]. Some studies have also found that having a large number of groups compensates for the negative effects of small group sizes [39, 41]. For example, Clarke and Wheaton [41] found in their simulation study that when number of groups is greater than 150 the bias resulting from even extremely small group sizes is considerably reduced. In our data, we have a total of 60 clusters with size varying from 3 and 80 individuals, with a mean of 38. To minimize bias in the random components of the estimates, we follow Clarke & Wheaton [41] and limit the analyses in this manuscript to respondents residing in clusters with at least ten individuals. This approach resulted in a loss of five groups and 29 individuals. The final sample used for the analysis comprised 2,269 individuals. We estimated the models using the *gllamm* command in Stata 13 (College Station, Texas, USA).

### Ethical approval and informed consent

The Johns Hopkins Bloomberg School of Public Health Institutional Review Board (IRB) and the University of Liberia, Pacific Institute for Research and Evaluation IRB approved this survey. Only adults were enrolled into the study. Interviewers obtained verbal informed consent from all participants prior to the interviews. The researchers opted for verbal consent due to the low literacy level of a significant proportion of the study participants. The person obtaining the consent signed the consent document to attest that consent had been obtained. The two ethics committees approved this procedure.

## Results

### Socio-demographic characteristics of households and members

Table 1 provides information on the socio-demographic and ideational characteristics of the population in households with at least one ITN. Almost half (47.3%) of the population were adults, and household members were equally divided between males and females. In terms of female caregiver’s characteristics, most of the study population came from households where the female caregiver had little or no education. The ideational characteristics of the female caregivers revealed that fewer than one third (29.8%) of these women believed ITN use to be the norm among other households in their community. Nonetheless, the majority of the women believed in their susceptibility to malaria and perceived malaria to be a serious disease. Furthermore, most of the caregivers had a high level of perceived self-efficacy to prevent malaria and to detect a serious case of malaria, although the prevalence of comprehensive knowledge about malaria was low. More than half of the female caregivers (57.5%) had a high level of exposure to the “Take Cover” communication campaign.

**Table 1:** Background characteristics of people in households with at least one ITN, Liberia 2014.

Variable	N	%
Age Group		
0–4 years	611	26.9
5–17 years	584	25.8
Adult	1074	47.3
Sex		
Male	1123	49.5
Female	1146	50.5
Female caregiver’s level of education		
None	1272	56.1
Primary	754	33.2
Secondary and above	243	10.7
Female caregiver’s perceived norm about ITN use in their community		
Perceived ITN use not to be the norm	1593	70.2
Perceived ITN use to be the norm	676	29.8
Female caregiver’s perceived susceptibility to malaria		
Low	519	22.9
High	1750	77.1
Female caregiver’s perceived severity of malaria		
Did not perceive severity	732	32.3
Perceived severity	1537	67.7
Female caregiver’s knowledge about malaria prevention		
Low	1780	78.4
High	489	21.6
Female caregiver’s perceived self-efficacy to detect a complicated case of malaria		
Low	722	31.8
High	1547	68.2
Female caregiver’s perceived self-efficacy to prevent malaria for self and children		
Low	440	19.4
High	1829	80.6
Female caregiver’s exposure to “Take Cover” malaria prevention communication		
Low	964	42.5
High	1305	57.5
Household wealth quintile		
Lowest	245	10.8
Second	274	12.1
Middle	482	21.2
Fourth	586	25.7
Highest	685	30.2
Number of under-5 children in household		
One	1250	55.1
Two	671	29.6
Three or more	348	15.3
Number of ITNs in household		
One	1209	53.3
Two	681	30.0
Three or more	379	15.7
County of residence		
Bong	735	32.4
Cape Mount	604	26.6
Grand Kru	601	26.5
Rivercess	329	15.4
**Number of people in households with at least one ITN**	**2269**	

More than half (54.7%) of the members were from households with only one under-five child (55.1%), and most were from households with only one (53.3%) or two (30.0%) ITNs. Proportionally fewer of the members were from the lowest or second wealth quintiles than from any of the three upper quintiles. Similarly, proportionally fewer of the household members were from Rivercess County compared to any of the other three counties.

### Patterns of ITN ownership, conditions, and use

A little over one third (36%) of surveyed households had at least one net. Almost all (99.1%) of these nets were ITNs; the rest were untreated nets. Overall, only 4% of the study households had enough ITNs for all household members as defined by the WHO as one ITN per two household members. Upon inspection, about three-fifths (58.3%) of the ITNs in use in the surveyed households were found to have no holes while 16.7% had very small holes (smaller than a flashlight battery); the rest (25%) had at least one hole larger than a flashlight battery. Most of the ITNs (78.7%) had been in the household for more than six months. In households with at least one ITN, two thirds (66.6%) of the members slept under an ITN on the night before the survey. This indicator varied widely by country: 85.6% in Bong, 76.3% in Cape Mount, 62.0% in Rivercess, and 36.3% in Grand Kru. A little over one tenth (12%) of the ITNs within the households were not used on the night prior to the survey. Nonetheless, in many households with ITNs, up to four persons slept under the same ITN; the median number of users per ITN was three household members.

### Correlates of intra-household ITN use

We used multilevel modeling to assess the correlates of intra-household ITN use. The results of the three-level models are presented on Table 2. The empty model revealed significant clustering of ITN use at both the household and cluster levels. The intra-class correlation (ICC) indicates that about 24% of the variance in intra-household ITN use is attributable to factors operating at the cluster level while about 60% of the variance is due to factors operating at the household level. This result justifies the use of multi-level modeling.

**Table 2:** Results (odds ratio) of the multilevel modeling of the relationship between ITN use and selected individual, household, and community variables, Liberia 2014.

Correlates	Empty Model^1^	Full Model^2^
***Fixed Effects***		
*Household member characteristics*		
Age Group		
0–4 years (RC)	---	1.00
5–17 years	---	0.090***
Adult	---	0.428**
Sex		
Male (RC)	---	1.00
Female	---	0.772
Interactions Sex/Age group		
Male X Age group 0–4 (RC)	---	1.00
Female X Age group 5–17	---	1.828‡
Female X Adult	---	2.783**
*Female caregiver’s socio-demographic and ideational characteristics*		
Female caregiver’s level of education		
None (RC)	---	1.00
Primary	---	0.978
Secondary and above	---	0.739
Female caregiver’s perceived norm about ITN use in the community		
Perceived ITN use not to be the norm	---	1.00
Perceived ITN use to be the norm	---	0.996
Female caregiver’s perceived susceptibility to malaria		
Low	---	1.00
High	---	0.841
Female caregiver’s perceived severity of malaria		
Did not perceive severity	---	1.00
Perceived severity	---	1.621*
Female caregiver’s knowledge about malaria prevention		
Low	---	1.00
High	---	1.323
Female caregiver’s perceived self-efficacy to detect a complicated case of malaria		
Low	---	1.00
High	---	2.044**
Female caregiver’s perceived self-efficacy to prevent malaria for self and children		
Low	---	1.00
High	---	0.957
Female caregiver’s exposure to “Take Cover” malaria prevention communication		
Low	---	1.00
High	---	1.902**
*Household characteristics*		
Household size	---	0.678***
Household wealth quintile		
Lowest (RC)	---	1.00
Second	---	1.301
Third	---	1.919‡
Fourth	---	1.766
Highest	---	2.467*
Number of under-5 children in household		
One (RC)	---	1.00
Two	---	1.067
Three or more	---	0.650
Number of ITNs in household		
One (RC)	---	1.00
Two	---	6.862***
Three or more	---	13.330***
*Community characteristics*		
County of residence		
Bong (RC)		1.00
Cape Mount		0.654
Grand Kru		0.179***
Rivercess		0.476*
***Random Effects***		
Cluster level variance (SE)	1.874***(.570)	0.005 (.119)
Cluster-level ICC^3^	.241	0.001
Household level variance	2.629***(.434)	1.981***(.37993)
Household-level ICC^3^	.596	.375
Log likelihood	-1144.69	-948.91
AIC	2297.39	1955.83
Number of observations	2269	

Notes

‡ p<0.1

* p<0.05

** p<0.01

*** p<0.001.

^1^ Model with no covariates

^2^ Model with covariates

^3^ Intra-class correlation

When the covariates are introduced (Full Model), the residual ICC was substantially diminished at the cluster level, indicating that much of the cluster-level variance was due to differences across communities in the covariates included in the estimated model. In contrast, there remains a significant level of clustering at the household level, indicating that irrespective of socio-demographic characteristics, there are inter-household differences in ITN use among households within the same cluster. The results of this model further reveal that the strongest correlates of intra-household ITN use are number of ITNs in the household, age, household size, and county of residence. Other significant correlates of ITN use include caregiver’s exposure to malaria prevention messages, her perceived self-efficacy to detect complicated malaria, her perceived severity of malaria, and household wealth.

There is a significant interaction between sex and age group, indicating that the relationship of sex with ITN use varies by age. The findings indicate that sex does not make a difference for ITN use among children under five. Sex does not significantly moderate the association of being an older child or teenager with ITN use. In contrast, among adults, being female significantly alleviates the negative effects of age on ITN use. Among male members, older children and teenagers were 91% less likely and adults were 53% less likely than under-five children to use an ITN. Furthermore, the predicted marginal probability from the estimated full model reveal that adult women (75.8%) were significantly more likely than adult men (65.7%) to sleep under an ITN: (p<0.001). For under-five children, the predicted marginal probability was 76.2% for boys and 76.3% for girls.

The number of ITNs in a household was one of the strongest predictors of ITN use; having three or more ITNs in a household is associated with a 13-fold increase in the odds of sleeping under an ITN. Individuals residing in households with a female caregiver who reported a high level of perceived severity of malaria were 62% more likely than their peers from other households to sleep under an ITN. Similarly, a female caregiver’s high level of perceived self-efficacy to detect complicated malaria was associated with a two-fold increase of household members’ use of ITNs. The relationship with exposure to the “Take Cover” campaign was such that individuals from households in which the female caregiver had a high level of exposure were 90% more likely to sleep under an ITN compared to individuals from households in which the female caregiver had a low level of exposure.

The relationship with household size is negative, such that a unit increase in household size is associated with a 32% reduction in the odds of using an ITN. As for household wealth, the only significant difference is between the lowest and highest quintiles, with individuals from the highest quintile being more than twice as likely to sleep under an ITN as their peers from the lowest quintile. Individuals from Grand Kru and Rivercess were significantly less likely to sleep under an ITN than their counterparts from Bong. Finally, the number of under-five children in the household was not significantly associated with intra-household ITN use.

## Discussion

In this manuscript, we examine the correlates of ITN use among household members in four counties in Liberia using multilevel modeling of survey data. The data showed that only about a third of households had at least one ITN, and fewer households still had universal coverage. Nonetheless, in households with at least one ITN, about two-thirds of the members slept under an ITN on the night preceding the survey.

Results of the multilevel model revealed that the strongest correlates of intra-household ITN use are number of ITNs in the household, age, household size, county of residence, female caregiver’s ideational characteristics, her exposure to malaria prevention messages, and household wealth. These findings echo what extant literature already tells us, while also providing new evidence. For example, consistent with evidence from previous studies, we found that the odds of ITN use were considerably lower among older children and adults compared to under-five children [16–21, 42]. Moreover, we found that being a female alleviates the negative correlation of older age with ITN use. Both of these findings indicate that people understand and tend to act upon the message that young children and pregnant women are the most vulnerable to malaria. Consistent with what other studies have found [26], the findings also suggest that the woman (rather than the couple) is the one most likely to share a sleeping space with her young children.

This study found that certain female caregiver’s ideational characteristics are associated with ITN use among members of her household. The association between a female caregiver’s ideational characteristics and experiences of her household members echoes evidence from other health domains including family planning [35–36], HIV [37], water and sanitation [43], and female genital mutilation [44]. The positive association of female caregiver’s perceived severity of malaria with household members’ ITN use is consistent with evidence from prior studies [45–47]. Female caregiver’s perceived self-efficacy to recognize a complicated case of malaria was positively associated with ITN use. While evidence concerning the role of this specific variable is rare in literature, the finding is consistent with what some studies have found regarding the link between malaria knowledge and ITN use [23, 30–32]. In contrast to some prior studies [45–47, 48–49], this study did not find any significant relationship between perceived susceptibility to malaria and use of ITNs. While the reason for this finding is not clear, it is possible that use of ITNs tends to suppress perceived susceptibility to malaria, as ITN users perceive themselves to be protected against malaria. Indeed, a few studies have shown a negative relationship between perceived susceptibility to malaria and use of an ITN [23, 46].

This study revealed a positive association between exposure to malaria-prevention communication messages and use of ITNs, echoing findings from other studies [17, 23, 28, 48]. Furthermore, consistent with previous studies, we found a positive link between household wealth and ITN use [18, 24]. The strong association of the number of ITNs in the household and ITN use makes intuitive sense and has been found in other studies [31]. The significantly lower ITN use in Grand Kru and Rivercess compared to Bong is due to the fact that considerably fewer households in these two counties had enough ITNs and indicates that these two counties should be prioritized in ITN distribution efforts.

Finally, after controlling for measured variables at the individual, household, and community levels, there are significant unmeasured variables operating at the household level that influence ITN use. While this study did not allow us to identify the specific factors, it is possible that they include socio-demographic and ideational characteristics of the male head of household, conjugal power dynamics, sleeping arrangements (defined by the number, type and location of sleeping spaces, number of persons per sleeping space, and ability to hang an ITN over sleeping spaces), and recent experience of fever among members of the household. Indeed, it is reasonable to assume that male heads of household, as the key decision-makers, exert great influence on health behavior decisions within the household. The position of male heads of household on ITNs, determined by their socio-demographic and ideational characteristics, can be expected to influence household use of ITNs. In the same vein, non-egalitarian conjugal power that puts the woman at a disadvantage in household decision-making may limit her influence on household use of ITNs even if she possesses relevant positive ideational characteristics. Furthermore, a recent episode of malaria may increase a person’s desire for malaria prevention and therefore increase ITN use. Indeed some studies have found a positive relationship between recent experience of malaria and willingness to pay for ITNs [50]. Sleeping arrangements vary across households and have been found to affect ITN use [51, 52].

The findings of this study have important implications for programming, policy, and further research. In general, the findings underscore the need for health communication program management to conduct thorough audience profiling in order to construct targeted and effective messages. The considerably lower likelihood of men, older children, and teenagers to use an ITN is concerning. Two interrelated reasons may be adduced for this finding: insufficient ITNs within the households leading to prioritization of allocation for the household members perceived to be most vulnerable; and attitudinal (for example, the belief that malaria is a less serious problem for men and older children and therefore use of ITNs is less important for them). Our study has shown a strong relationship between the number of ITNs in the household and the odds of sleeping under an ITN. The implications of this finding are obvious- helping households attain universal coverage will reduce discrepancies in intra-household ITN use. Since 2010, universal coverage has been a global goal for malaria-endemic countries. To this end, Liberia carried out a universal coverage campaign in April 2015 and is currently scaling up ITN distribution via antenatal care visits and other channels. As many studies have demonstrated, ITN use increases significantly following ITN distribution programs [22, 53–64]. Future evaluations will hopefully confirm this for Liberia.

In addition, the documented importance of ideational constructs (specifically female caregiver’s perceived severity of malaria and perceived self-efficacy to recognize a complicated case of malaria) for ITN use among household members underscores the relevance of an ideational model in developing malaria prevention programs. These programs should seek to increase audience understanding of the severity of malaria and improve knowledge about the symptoms of complicated malaria. Furthermore, the wealth inequity in use of ITNs suggests the need to ensure that poor households are not overlooked in ITN distribution strategies, and that they receive messages to motivate usage of ITNs.

The finding of residual clustering at the household level indicates that interventions that specifically target households are relevant. Such interventions can target heads of households with audience specific malaria-prevention information, motivational messages, and skills to enable them take health-protective decisions for their families. Peer education interventions that train and equip household heads with relevant materials, mobilize them to reach out to their peers in the community to educate them about ITNs, and encourage them to use ITNs are also relevant.

This study has some limitations that warrant mention. First, the data analyzed are derived from a cross-sectional survey, which precludes all attempts at causal attribution. The relationships documented in this manuscript are associations, although the magnitude of the relationships implies that their implications should be taken seriously. Secondly, since the data are self-reported, they are subject to social desirability bias and memory lapse. Nonetheless, we believe that the rigorous data collection practices during fieldwork have helped to minimize these problems.

## Conclusion

The factors associated with ITN use in Liberia operate at multiple levels, including individual, household, and community. Programs should ensure universal coverage of ITNs and encourage all household members to sleep under them. Interventions designed to promote ITN use are likely to be effective if they address barriers at the individual, household and community levels. Programs should seek to identify and evaluate ideational factors when designing ITN use promotion campaigns. This study indicates that a better understanding of household level factors such as conjugal power dynamics and ideational characteristics of male heads of household is needed to effectively encourage intra-household ITN use in Liberia.
